# Review of the Problems at the Quarterly Court

**Published:** 1914-12-05

**Authors:** 


### THE WAR AND THE LONDON HOSPITAL.

### Review of the Problems at the Quarterly Court.

The Quarterly Court of the London Hospital was held on Wednesday afternoon, Viscount Knutsford presiding, and we take the following matters of interest from the adopted report:—

### Half-pay for Those on Active Service.

The Committee has decided to give half-pay to all such of the hospital employees as have joined the Army as Reservists or have enlisted in Kitchener's Army. The money is to be paid to the person nominated by the employee, and their posts are to be kept open for them on their return. In the case of medical and surgical "employees," such as the registrars, it was decided that they should be paid the difference between their salaries in the Army and the salary they earn at the hospital, so that they should be at no loss in taking up an Army appointment. Should they be earning in the Army as much as or more than would be paid to them at the hospital, then no salary is to be paid to them, but their appointments are to be kept open. Up to the present time forty-three members of the lay staff and sixty-two qualified doctors have joined H.M. Forces from the London Hospital. The latter figure includes surgeons and physicians, assistant surgeons and assistant physicians, medical and surgical registrars, house physicians and house surgeons. A large number of students also have joined the Royal Army Medical Corps as dressers and carriers.

Arrangements have been made to ensure that there should be no suffering on the part of the civilian patients on account of the departure of so many members of the staff. Most of those who are left are voluntarily doing almost double work, and it must also be remembered that nearly all teaching has stopped, and that little or no research work can be carried on. The hospital, during the war, has returned to its elementary duties in giving attention for the relief of those who are at the moment in its beds. Any further members of the visiting staff who wish to go to the Front will only be allowed to do so after the Committee has given the subject very careful consideration, as it is felt that the staff of the hospital has now reached the smallest numbers under which the work can be satisfactorily carried on.

### The London Hospital Contingent for France.

The Committee has had under consideration for some time the desirability of dispatching to France a London hospital contingent, fully staffed by surgeons, physicians, nurses, dressers, radiographers and others. Should the War Office desire such a contingent it will be immediately forthcoming. By such an arrangement, most members of the hospital staff would be able to take a term of service with this contingent, and it is felt that it would be much more satisfactory for them than to join various private units for variable periods.

The hospital so far has admitted from 1,300 to 1,400 wounded soldiers, and of these 400 or 500 have been Belgians. Only five have died, and as some of the wounds were very grave indeed, the hospital service at the Front and the treatment the men had received at the field hospitals are seen to be excellent.

While the hospital has offered 250 beds to the Army and 250 to the Navy, it has never had more than 400 wounded in at the same time. No seriously ill civilian case has ever been sent away because of the presence of the wounded. It is true that non-urgent civilian cases, cases which can very well wait without suffering, have been added to the waiting list, but the report remarks that no suffering has been caused thereby.

### Playing at Trenches.

Apart from numerous Royal and distinguished visitors, the hospital has been fortunate in that it has received most generous gifts on behalf of the wounded—viz. game, fruit, cigarettes, articles of clothing, games, books, etc. A large present of plasticine gave great delight, and competition was organised and prizes given for the best forms of entrenchment: in many of the model trenches made by the soldiers the greatest ingenuity was shown. Some of them made exact models of the trenches in which they had been wounded. Captain Fenwick gave first, second, and third prizes for these models. One gentleman sent £50 for the purchase of warm clothes for soldiers after they leave the hospital.

### The Hospital and Contractors.

When the war broke out contractors were informed, with regard to their contracts, that if they would continue the supply of goods, any application they might make to the House Committee for extra charges would be carefully and sympathetically considered. Such applications were made and were carefully considered by the Steward's Committee as well as the House Committee, and the total sum asked for by the contractors over and above contract prices during the first three months of the war came altogether to £351 17s. 11d. The Committee considered each of the requests made most reasonable, and gladly acknowledge the fair way in which the hospital has been treated by firms who have dealt with it. Almost without exception firms who supply meat, milk, bread, groceries, drugs, etc., have shown an extremely friendly spirit to the hospital.

In no department has the result of the war been more plainly noticed than in the drug department. Many of the drugs commonly used are made in Germany, and others are affected indirectly or directly by the war. Such an important drug as carbolic acid, for instance, has advanced from our contract price of 3¾d. to 1s. 4d. per lb. This, no doubt, is due to the demand for it for the manufacture of explosives. Fortunately, the hospital has more than six months' supply in stock.

Christmas Festivities Cancelled.

The Committee have decided to cancel all Christmas festivities at the hospital this year. These festivities at the London Hospital are, of course, rather a feature—stages are put up in ten or twelve of the wards, resident doctors organise themselves into various troupes, outside entertainers lend their assistance, Father Christmas and his fairies visit all the wards, special Christmas fare is given to all patients well enough to enjoy it, and carols are sung by nurses in the early morning.

This year, unfortunately, the resident doctors who—as has been stated—are nearly all doing double work, and, moreover, are constantly changing, have no time to get up Christmas troupes; neither have the lay staff any leisure to arrange for the putting up of stages, etc. Moreover, it is not in the least unlikely that quite a large number of wounded might be received on Christmas Day itself, for they come at short notice night and day. However, Sir Edgar Speyer has once again kindly agreed to give a present to every patient in the hospital on Christmas Day, and these presents will be quietly given out by the sisters of the wards. Probably "Punch and Judy" will be engaged for the children. Christmas fare will, of course, be given, and the nurses intend to give their usual carols.

